# A comprehensive genome‐wide analysis of long noncoding RNA expression profile in hepatocellular carcinoma

**DOI:** 10.1002/cam4.1180

**Published:** 2017-10-18

**Authors:** Hongxia Cui, Yunxing Zhang, Qiujie Zhang, Wenming Chen, Haibo Zhao, Jun Liang

**Affiliations:** ^1^ Department of Medicine Qingdao University Qingdao China; ^2^ Department of Oncology Jining First People's Hospital Jining China; ^3^ Department of Emergency trauma surgery Jining First People's Hospital Jining China; ^4^ Department of Oncology The Peking University International Hospital of Peking University Beijing China

**Keywords:** Biomarker, HCC, lncRNA, metastasis, SNHG7

## Abstract

Hepatocellular carcinoma (HCC) is one of the most common malignancies worldwide, especially in East Asia and China. Long noncoding RNAs (lncRNAs) are emerging as critical regulators that may be involved in the development and progression of cancers in humans. However, the contributions of lncRNAs to HCC development, metastasis, and recurrence remain largely unknown. In this study, we comprehensively investigated lncRNA expression profile in HCC and normal tissues using TCGA RNA sequencing data, one RNA sequencing dataset, and two microarray datasets from GEO. By analyzing these four datasets, we identified hundreds of expression‐dysregulated lncRNAs in HCC tissues compared with normal tissues. Genomic copy number variation analysis showed that many of those lncRNAs disorder are related to the copy number amplification or deletion. Moreover, several lncRNAs expression levels are associated with HCC patients' overall and recurrence‐free survival, such as RP1‐228H13.5, TMCC1‐AS1, LINC00205, and RP11‐307C12.11. Furthermore, we identified two lncRNAs termed PVT1 and SNHG7 that may be involved in HCC cells metastasis by comparing lncRNAs expression profiles between early recurrence HCC tissues with metastasis and late recurrence HCC tissues without metastasis. Finally, loss‐of‐function assays confirmed that knockdown of SNHG7 and PVT1 impaired HCC cells invasion. Taken together, these findings may provide a valuable resource for further identification of novel biomarkers and therapeutic targets for HCC patients.

## Introduction

Hepatocellular Carcinoma (HCC), a highly aggressive malignancy, is one of the most common cancers and represents the second leading cause of cancer‐caused death worldwide 1. In spite of the improvement in diagnostic technology, surgical therapy, chemotherapy, and molecular targeting therapy, the overall 5‐year survival rate of HCC patients still remains unsatisfied due to the recurrence and metastasis 2, 3. Although some important driver genes have been uncovered in HCC, the mechanism of HCC development, metastasis, and recurrence have not yet been well clarified 4, 5. Therefore, a better understanding of the biological mechanisms involved in HCC pathogenesis and progression is critical for the developing of novel diagnostic biomarkers and therapeutic strategies.

In the past decade, advances in the high‐throughput RNA sequencing techniques and bioinformatics methods have led to the identification that less than 2% of whole human genome sequence is protein coding gene, while the majority of the genome is transcribed into noncoding RNAs such as microRNAs, and long noncoding RNAs (lncRNAs) 6, 7, 8. lncRNA is a novel member of ncRNA, which is longer than 200 nt in length and lacks protein coding capacity. Increasing evidence have revealed that lncRNAs are widely expressed in human tissues, and participate in many important cellular biological processes, such as cell fate decision, stem cells differentiation, X chromosome imprinting, cancer cells metastasis, and drug resistance 9, 10, 11. Importantly, it has been reported that lots of lncRNAs dysregulation contributes to several diseases development 12. In addition, analyses of several large‐scale cancer tissue samples' RNA sequencing and microarray datasets have uncovered that lots of lncRNAs showed altered expression pattern in multiple human cancers, and many of these lncRNAs could affect cancer cells phenotype via repression of tumor suppressors' expression or activation of oncogenes transcription 13, 14, 15. For example, overexpressed gastric cancer‐associated lncRNA HOXA11‐AS promotes cell proliferation and invasion through repressing KLF2 and PRSS8 transcription through functioning as a scaffold of EZH2, DNMT1, and LSD1 16.

Recently, the biological roles of lncRNAs have been frequently documented in HCC development and progression. For example, lncRNA FTX is down‐regulated in HCC tissues, and it could inhibit HCC cells invasion and proliferation by repressing Wnt/*β*‐catenin signaling activity and impeding DNA replication through competitively sponging miR‐374a and binding to the DNA replication licensing factor MCM2 17. In addition, overexpressed lncRNA unigene56159 promotes HCC cells migration and invasion through binding to miR‐140‐5p and effectively acting as a ceRNA for miR‐140‐5p to derepress the SNAI2 expression 18. Although functions and mechanisms of some lncRNAs have been characterized in HCC, the expression pattern and relevance of the majority lncRNAs in HCC remain unknown. To identify HCC‐associated lncRNAs, we investigated lncRNAs profiles in HCC and adjacent nontumor samples by analyzing TCGA RNA sequencing data and GEO RNA sequencing and microarray datasets. This study reveals the aberrant lncRNAs expression in HCC tissues, which may provide new potential candidates for HCC diagnosis and treatment.

## Methods and Materials

### TCGA and public microarray datasets analyses

The RNA sequencing data and corresponding clinical data from TCGA liver hepatocellular carcinoma and paired normal tissues were downloaded from the GDC Data Portal (https://portal.gdc.cancer.gov). Another four HCC public datasets (GSE77509 19, GSE64041 20, GSE70880 21, and GSE67260 22) were downloaded from Gene Expression Omnibus (GEO). Gene expression profiling of GEO microarray dataset GSE64041 was analyzed based on the Affymetrix Human Gene 1.0 ST Array platform, GSE77509 dataset was analyzed based on the Agilent‐038314 CBC Homo sapiens lncRNA + mRNA microarray V2.0 platform, and GSE67260 dataset was analyzed based on the Agilent‐052909 CBC_lncRNAmRNA_V3 platform. For TCGA differential gene expression analysis, Mann–Whitney U test was applied to the log2‐trnasformed upper‐quartile normalized FPKM values (from GDC Data Portal). For GEO Microarray datasets, the microarray probes were reannotated to lncRNAs, and the R package, limma, was used to calculate the differentially expressed lncRNAs.

### Copy number variation analysis

The HCC copy number variation data were downloaded from Broad GDAC FireBrowser website (https://portal.gdc.cancer.gov). GISTIC 2.0 23 was used to determine significantly recurrent lncRNAs genomic regions of somatic copy number alterations (SCNA). The amplification and deletion peaks with *q*‐values < 0.25 were considered as significant. The lncRNAs regions were mapped to the GISTIC peaks.

### lncRNAs Survival analysis

The univariable Cox regression analysis was used to determine the relationship between each lncRNA expression and HCC patients' overall survival (OS) and recurrence‐free survival (RFS). Only the lncRNAs with the *P* < 0.05 were considered statistically significant. Then, the HCC patients were divided into high‐ and low‐expression groups according to the median lncRNAs expression level. Log‐rank test was applied to compare the survival distribution between two groups. The Python package, lifelines, was used for the survival analysis.

### Cell culture and transfection

HCC cell line HuH1 was purchased from the Type Culture Collection of the Chinese Academy of Sciences (Shanghai, China). HuH1 cells was cultured in Dulbecco's modified Eagle's medium (Invitrogen, Carlsbad, CA) containing 100 U/mL penicillin, and 1 *μ*g/mL streptomycin (Invitrogen), 10% fetal bovine serum (Invitrogen, Shanghai, China) at 37°C with 5% CO_2_. The SNHG7, PVT1, and negative control siRNAs (Invitrogen, Carlsbad, CA) were transfected into HuH1 cells using RNAiMAX (Invitrogen) according to the manufacturer's instructions. Forty‐eight hours post transfection, the cells were harvested for RNA extraction. The SNHG7 siRNA sequences are siRNA 1#, GGAACAGGGTCAATCCTCCAATGTA, siRNA 2#, CCAGTCGGTCTTGTGTTCACATTGA. The PVT1 siRNA sequences are siRNA 1#, GCUGAGAGGGUUGAGAUCUCUGUUU, siRNA 2#, UGGACAGUCUGUGGCUGGGUGGGAA.

### RNA extraction and qPCR

RNA was extracted from HuH1 cells using TRIZOL reagent, according to the manufacturer's instructions. One microgram of total RNA was reverse transcribed into cDNA using PrimeScript RT Reagent Kit (Takara, Dalian, China). To examine SNHG7 and PVT1 expression levels, SYBR Premix Ex Taq (Takara) was used. The housekeeping gene GAPDH was used as an internal control. The primer sequence of SNHG7 is, forward 5′‐GTGTGTCCCTTGGTGGAGAG‐3′, reverse 5′‐TCCCAGATACCAGCGAAGGA‐3′. The primer sequences of PVT1 are: forward 5′‐TGAGAACTGTCCTTACGTGACC‐3′; reverse 5′‐AGAGCACCAAGACTGGCTCT‐3′. The primer sequences of GAPDH are: forward 5′‐AGAAGGCTGGGGCTCATTTG‐3′; reverse 5′‐AGGGGCCATCCACAGTCTTC‐3′. QPCR analyses were performed on ABI7500, and comparative cycle threshold (CT) (2^−ΔΔCT^) method was used to analyze the data.

### Cell migration and invasion assays

Transwell assays (Corning, Tewksbury, MA, USA, 8.0‐*μ*m pores) were performed to measure HuH1 cell invasive ability after transfection with SNHG7 or negative control siRNAs. Cells (1 × 10^5^) in 300 *μ*L medium containing 1% FBS were placed in the upper chamber of an insert (coated with Matrigel for invasion assay, Sigma‐Aldrich); 700 *μ*L medium with 10% FBS was added to the lower chamber. Twenty‐four hours after incubation, the HuH1 cells invaded through the membrane were fixed with methanol, then stained with 0.1% crystal violet, and imaged using an IX71 inverted microscope (Olympus, Tokyo, Japan).

### Statistical analysis

The one‐way ANOVA and Student's *t* test (two‐tailed) was used to analyze the in vitro data using R. A *P* < 0.05 was considered statistically significant.

## Results

### Identification of lncRNAs alterations in HCC tissues

**Figure 1:**
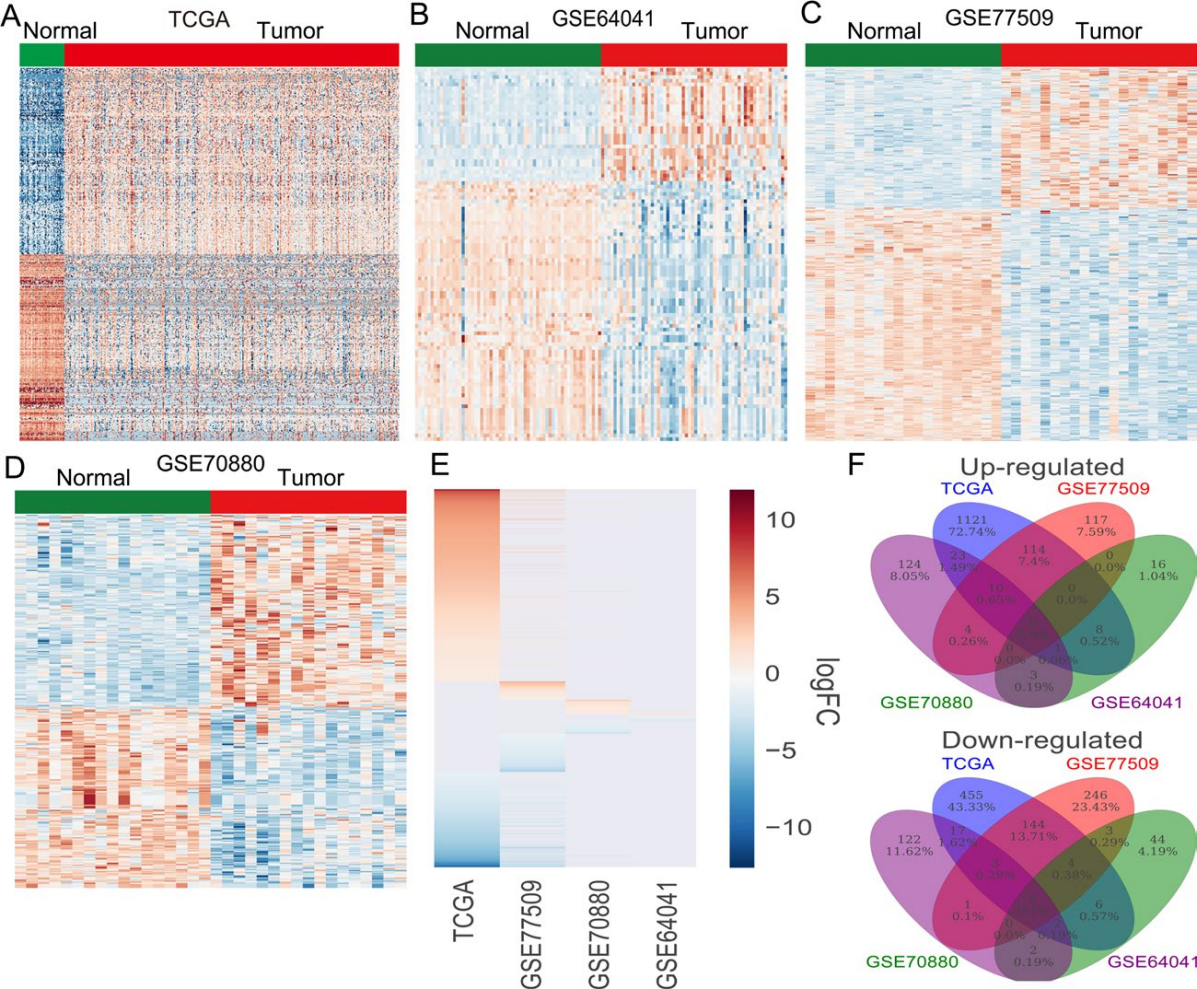
The lncRNAs expression profiling in HCC tissues and normal tissues. (A) A heatmap was drawn to show the altered lncRNAs expression profiles in HCC and normal tissue samples were analyzed using the TCGA datasets. (B‐D) A heatmap was drawn to show the altered lncRNAs expression profiles among HCC and normal tissues were analyzed using the GSE64601, GSE77509, and GSE70880 datasets. Red indicates increased relative expression and blue indicates decreased relative expression. (E) A heatmap was drawn to show the lncRNAs that consistently up‐regulated or down‐regulated in at least two datasets. (F) Venn diagram of differentially expressed lncRNAs in TCGA, GSE64601, GSE77509, and GSE70880 datasets.

To investigate the lncRNA expression profiles in HCC tissues, we used the RNA sequencing data and microarray gene profiling data (GSE77509, GSE64041, GSE70880) from TCGA and GEO in HCC and nontumor tissue samples. The TCGA project includes 374 HCC and 50 normal tissue samples, while the GSE77509, GSE64041, and GSE70880 datasets contains 20, 60, and 16 paired HCC and adjacent normal samples, respectively. Analyses of these datasets revealed that 1939 lncRNAs expression were dysregulated in the TCGA dataset (1295 up‐regulated and 644 down‐regulated); 657 lncRNAs was differentially expressed in the GSE77509 dataset (249 up‐regulated and 408 down‐regulated); 103 lncRNAs were dysregulated in the GSE64041 dataset (31 up‐regulated and 72 down‐regulated); and 335 lncRNAs were differentially expressed in the GSE70880 dataset (176 up‐regulated and 159 down‐regulated) (Fig. 1A–D, and Table S1). Further intersection analysis revealed that 347 lncRNAs were consistently up‐regulated or down‐regulated in at least two datasets (Fig. 1E and F). These findings indicate that a large number of lncRNAs have dysregulated expression levels in HCC, and some of these lncRNAs may be potential biomarkers for HCC diagnosis.

### Somatic copy number alterations of lncRNAs in HCC

**Figure 2:**
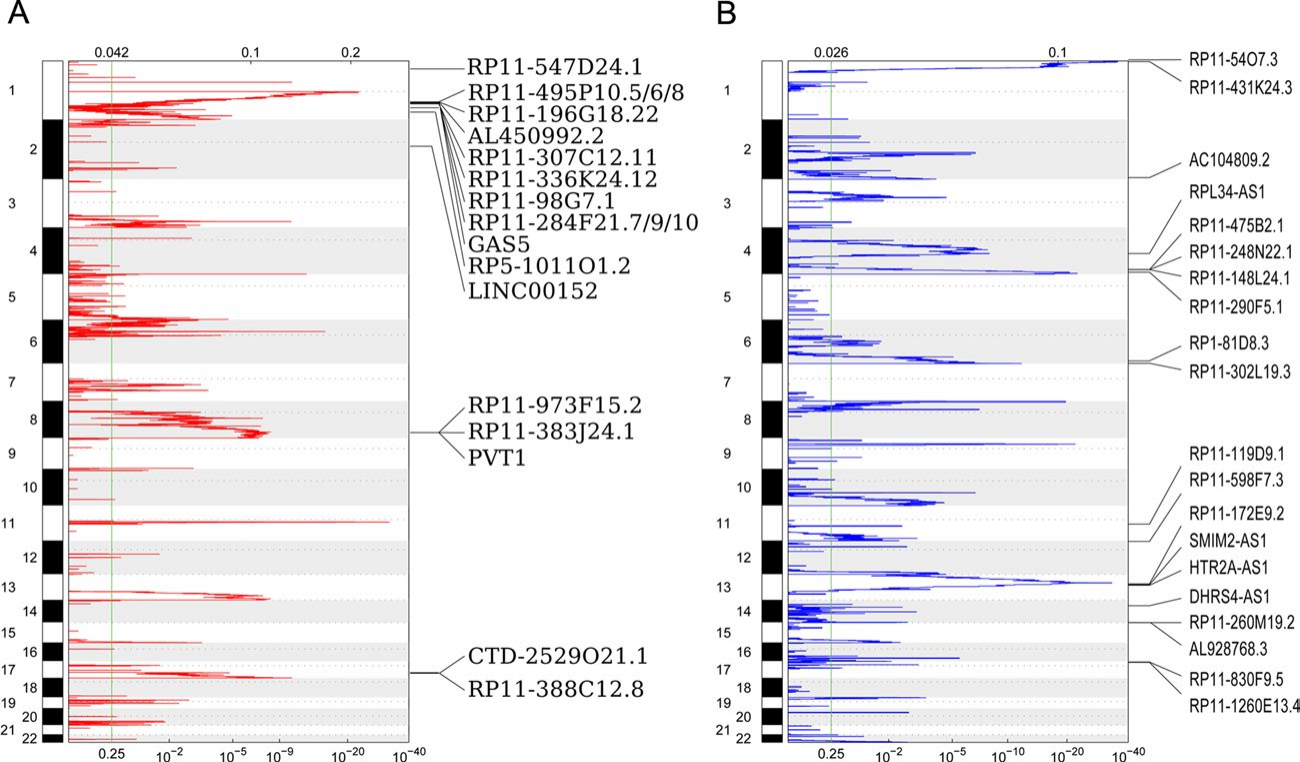
The Copy number gain and loss of dysregulated lncRNAs in HCC. (A) Frequency of lncRNAs copy number gain (red) in HCC tissues, and the rows are arranged according to the lncRNAs genomic locations, each of which represents a gene locus. (B) Frequency of lncRNAs copy number loss (blue) in HCC tissues, and the rows are arranged according to the lncRNAs genomic locations, each of which represents a gene locus. *X*‐axis presents the q‐value, and the green line represents the significance threshold (q‐value = 0.25).

To determine whether genomic alterations contribute to lncRNA dysregulation in HCC, we analyzed the copy number alterations of these differentially expressed lncRNAs (in TCGA data) using TCGA data. For HCC, the gene‐containing loci SCNAs frequencies of each lncRNAs were calculated. An alteration that occurs in all HCC samples with q value less than 0.25 was defined as significant alteration. The results showed 31 and 41 lncRNAs loci had recurrent DNA gain and loss, respectively (Fig. 2A and B, and Table S2). These findings suggest that genome copy number variations are involved in some lncRNAs dysregulation in HCC.

### Identification of survival‐related lncRNAs in HCC

**Figure 3:**
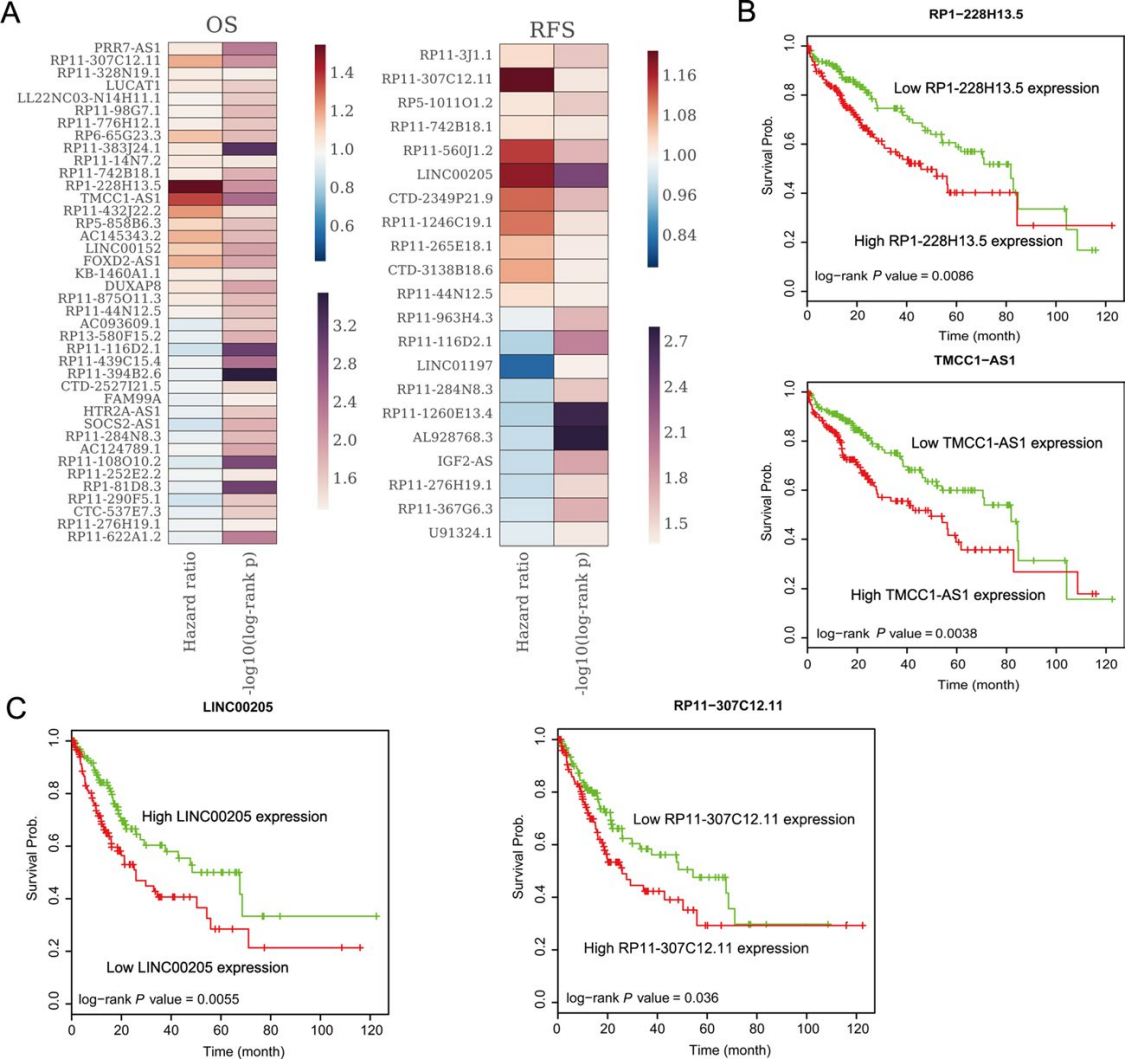
Hepatocellular carcinoma (HCC) Patients' overall survival (OS) and recurrence‐free survival (RFS)‐associated lncRNAs. (A) A heatmap was drawn to show the OS and RFS‐associated lncRNAs (p value, and Hazard ratio value were shown) in HCC. (B) The Kaplan–Meier curve for OS of HCC patients with high‐ RP1‐228H13.5 or TMCC1‐AS1 expression and low‐RP1‐228H13.5 or TMCC1‐AS1 expression using the TCGA data. The significant differences were examined using the two‐sided log‐rank test. (C) The Kaplan–Meier curve for RFS of HCC patients with high‐LINC00205 or RP11‐307C12.11 expression and low‐LINC00205 or RP11‐307C12.11expression using the TCGA data. The significant differences were examined using the two‐sided log‐rank test.

To evaluate whether the lncRNAs alterations are related with HCC patients' survival time, we performed univariable Cox regression analysis. The results revealed that 22 up‐regulated lncRNAs and 18 down‐regulated lncRNAs are significantly associated with HCC patients OS (*P* < 0.05), while 11 up‐regulated lncRNAs and 10 lncRNAs down‐regulation are significantly associated with HCC patients RFS (Fig. 3A, Table S2). For example, HCC patients with higher RP1‐228H13.5 and TMCC1‐AS1 expression levels had shorter OS time, while HCC patients with higher RP11‐307C12.11 and LINC00205 expression levels had shorter RFS time (Fig. 3B and C). These findings indicate that these HCC survival‐associated dysregulated lncRNAs could be used as biomarkers for HCC patients' prognostic prediction.

### Identification of lncRNAs alteration in HCC tissues with metastasis

**Figure 4:**
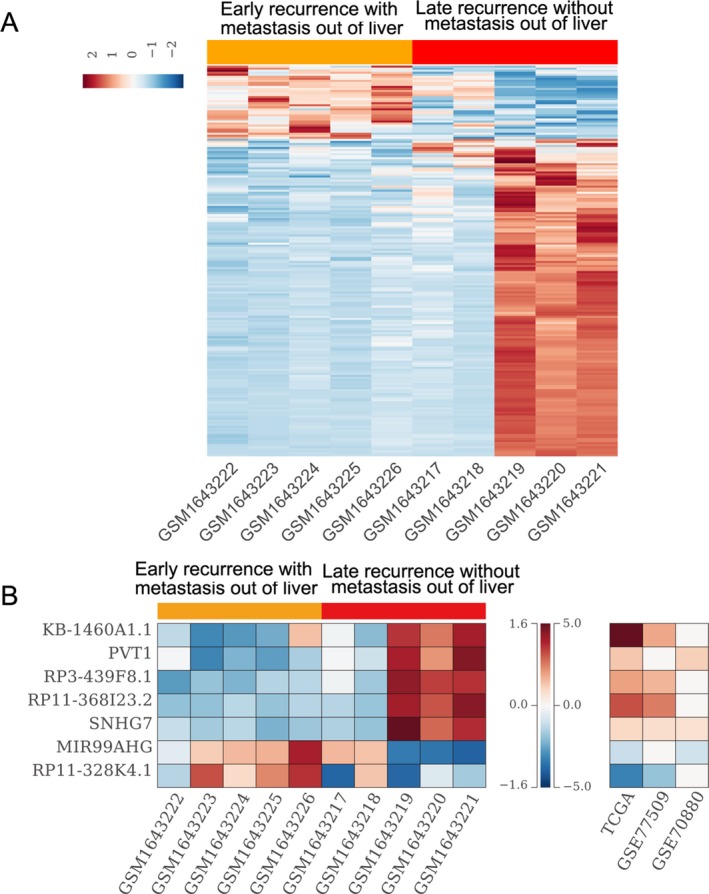
Differently expressed lncRNAs in HCC tissue with or without metastasis out of liver**.** (A) A heatmap was drawn to show the altered lncRNAs expression profiles in HCC tissue samples with early recurrence (less than 1 year, with invasion and metastasis out of liver) compared with that in HCC tissues with late recurrence (longer than 2 years, without invasion and metastasis out of liver) using GSE67260 dataset. (B) A heatmap was drawn to show the significantly up‐regulated or down‐regulated lncRNAs expression profiles in HCC tissue samples with metastasis, and their fold‐changes in HCC tissues compared to normal tissues in TCGA, GSE77509, and GSE70880 datasets.

Cancer cells invasion, metastasis, and tumor recurrence are few of the main reasons of HCC‐caused death. To identify lncRNAs that may be involved in HCC recurrence and metastasis, we downloaded the GSE67260 dataset from GEO, which consists of five tissue samples with early recurrence (less than 1 year, with invasion and metastasis out of liver) and five samples with late recurrence (longer than 2 years, without invasion and metastasis out of liver). Analysis of the data revealed that 156 lncRNAs expression were up‐regulated and 36 lncRNAs expression were down‐regulated in HCC samples with early recurrence when compared with the HCC tissues with late recurrence (Fig. 4A). Further intersection analysis showed that five up‐regulated lncRNAs in early recurrence samples are also overexpressed in HCC tissues compared with normal tissues, while two down‐regulated lncRNAs in early recurrence samples are also decreased in HCC tissues compared with normal tissues (Fig. 4B). These findings indicated that lncRNAs not only contribute to HCC development, but may also be involved in cancer cells metastasis and HCC recurrence.

### Knockdown of PVT1 and SNHG7 impaired HCC cells invasion

**Figure 5:**
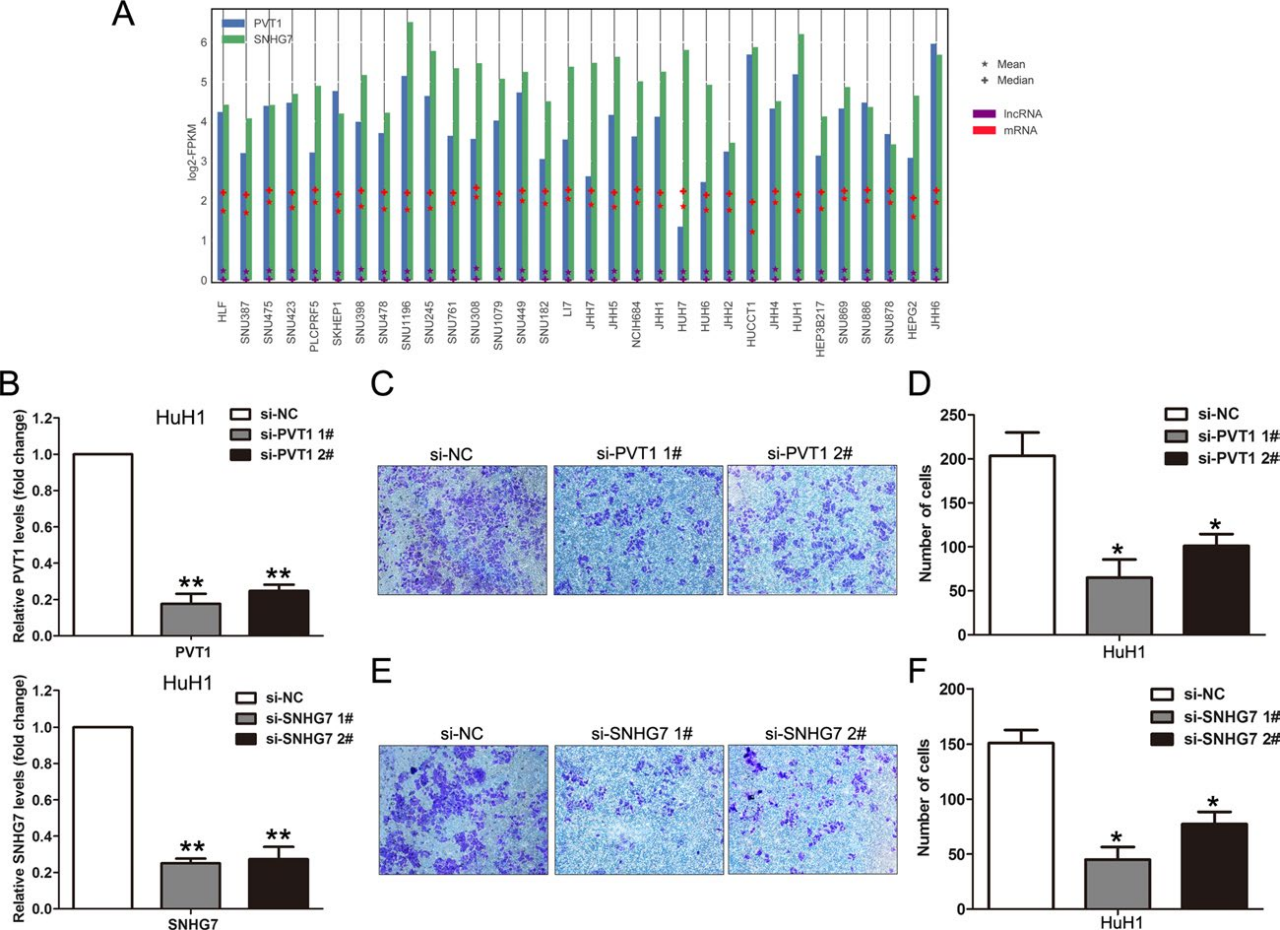
Down‐regulation of PVT1 and SNHG7 expression inhibited HCC cells invasion ability. (A) The expression levels of PVT1 and SNHG7 were detected in 28 HCC cell lines using Cancer Cell Line Encyclopedia (CCLE) RNA sequencing data. (B) qPCR analysis was used to examine the PVT1 and SNHG7 expression levels in HuH1 cells after transfection with PVT1 siRNAs, SNHG7 siRNAs, or negative control siRNAs. (C, D) Transwell assays were used to determine the invasive ability of si‐PVT1 or negative control siRNAs‐transfected HuH1 cells. Data represent the mean ± s.d. from three independent experiments. (E, F) Transwell assays were used to determine the invasive ability of si‐SNHG7 or negative control siRNAs transfected HuH1 cells. Data represent the mean ± s.d. from three independent experiments **P < 0.01; *P < 0.05.

To determine whether these lncRNAs could affect HCC cells invasion, we chose up‐regulated lncRNAs PVT1 and SNHG7 for further study. Recent studies reveal that PVT1 is overexpressed in multiple cancers and correlated with cancer progression, including colorectal cancer 24, gastric cancer 25, and cervical cancer 26. In addition, a recent study showed that SNHG7 overexpression promotes cell migration and invasion in lung cancer by enhancing the FAIM2 expression 27. Here, we firstly analyzed PVT1 and SNHG7 expression levels in HCC cell lines using CCLE data 28, and the results showed that HuH1 cell has highest PVT1 and SNHG7 expression levels (Fig. 5A). Then, we designed siRNAs for PVT1 and SNHG7, and transfected them into HuH1 cells to knockdown PVT1 and SNHG7 expression. The qPCR results showed that PVT1 and SNHG7 expression was significantly reduced in siRNA‐transfected cells compared with controls (Fig. 5B). Next, transwell assays revealed that knockdown of PVT1 and SNHG7 could impair HuH1 cells invasive ability compared with control cells (Fig. 5C–F). These results suggest that PVT1 and SNHG7 might play important roles in HCC invasion and progression.

## Discussion

In the past decade, increasing evidence have highlighted the critical roles of noncoding elements of human genomic in cellular process and their alteration in human diseases, such as cancer development and progression 29, 30. Recently, lots of studies have revealed the involvement of lncRNAs in tumorigenesis and cancer progression by functioning as oncogenes or tumor suppressors dependent on the circumstance. Moreover, high throughput RNA sequencing in diverse cancers and normal tissues has uncovered that some lncRNAs expressions pattern is tissue‐specific, and few cancer‐specific lncRNAs have been characterized in different cancers 8. These findings suggest that lncRNAs may be better biomarker candidates for human cancers diagnostic and prognostic prediction. However, the HCC‐associated lncRNAs and their function remain not well known.

In this study, we analyzed lncRNAs expression profiles in HCC tissues compared with parental normal tissues or nontumor tissues using TCGA RNA sequencing data, as well as RNA sequencing and microarray gene profiling datasets from GEO. Interestingly, our findings revealed that thousands of lncRNAs were differentially expressed in HCC tissues compared with normal tissues. Moreover, genomic alterations analyses showed that somatic copy number variations contribute to some of these lncRNAs dysregulation in HCC tissues. Importantly, survival analysis indicated that lots of lncRNAs overexpression or down‐regulation is significantly associated with HCC patients' poorer OS or RFS, which suggests that these lncRNAs could be valuable prediction factors for HCC patients' survival time. Cancer cells invasion, metastasis, and tumor recurrence are the main reason of HCC‐caused death. Here, we identified several lncRNAs that may be involved in HCC metastasis by comparing lncRNA expression pattern in HCC tissues with or without invasion and metastasis, such as PVT1 and SNHG7.

lncRNA PVT1 has been found to be up‐regulated in several cancers, and overexpressed PVT1 exerts oncogenic function by promoting cancer cells proliferation, invasion, and metastasis. For example, PVT1 is significantly up‐regulated in gastric cancer tissues, and increased PVT1 predicted poor prognosis. PVT1 promotes gastric cancer cell proliferation and invasion through directly binding with FOXM1 protein and increasing FOXM1 in a posttranslation regulation manner 25. In addition, Wan et al. reported that PVT1 is highly expressed in non‐small‐cell lung cancer tissues and cells, and enhances cells proliferation and inhibits apoptosis by interacting with EZH2 and repressing tumor suppressor LATS2 transcription 31. Besides, PVT1 could promote HCC cells proliferation and stem cell‐like property of HCC cells by stabilizing NOP2 32. Higher PVT1 expression level is associated with tumor progression and predicts recurrence in HCC patients 33. Tian reported that PVT1 promotes the tumorigenesis and metastasis of hepatocellular carcinoma by acting as a competing endogenous RNA for miR‐186‐5p 34, which is consistent with our analyses results. Moreover, our finding also reveals that knockdown of PVT1 impedes HCC cells invasion. Similarly, down‐regulation of another candidate SNHG7 also impairs cells invasion. SNHG7 overexpression has been found in lung cancer, and SNHG7 could promote lung cancer cells proliferation, invasion, and inhibit apoptosis by enhancing the FAIM2 expression 27. These findings suggest that our analyses results could provide valuable lncRNAs candidates for further investigation of lncRNAs function in HCC.

In summary, our findings uncover thousands of Dysregulated lncRNAs in HCC tissues compared with their parental normal tissues. Some of these lncRNAs are significantly associated with HCC patients' prognosis, and might play critical roles in HCC development, progression, and recurrence through regulation of cell invasion ability. Our study highlights the critical roles of lncRNAs in HCC and may provide valuable candidates as diagnostic markers and potential targets for HCC patients. This study also has a few limitations; for example, only a few lncRNA candidates were validated in this study. This will need to be further investigated by other researchers.

## Conflicts of Interest

No potential conflicts of interest were disclosed.

## Supporting information

Table S1. The lncRNAs profiling in TCGA and GEO datasets.

Table S2.

Table S3. The lncRNAs CNV, and survival associated lncRNAs in HCC.
